# Synthesis and Characterization of Antifungal Nanocomposite AgSiO _2_ Polymethyl Methacrylate

**DOI:** 10.1055/s-0041-1731831

**Published:** 2021-08-12

**Authors:** Mahmoud Sabouhi, Maryam Amini-Pozveh, Farshad Bajoghli, Hamid reza Dastjerd, Rasoul Mohammadi

**Affiliations:** 1 Department of Prosthodontics, Dental Materials Research Center, Dental Research Institute, School of Dentistry, Isfahan University of Medical Sciences, Isfahan, Iran; 2 Department of Prosthodontics Dentistry, Dental Materials Research Center, Dental Research Institute, School of Dentistry, Isfahan University of Medical Sciences, Isfahan, Iran; 3 Department of Prosthodontics, Dental Implants Research Center, Dental Research Institute, School of Dentistry, Isfahan University of Medical Sciences, Isfahan, Iran; 4 Institute of Biomaterial of Tehran University, Tehran University of Medical Science, Tehran, Iran; 5 Department of Medical Parasitology and Mycology, School of Medicine, Infectious Diseases and Tropical Medicine Research Center, Isfahan University of Medical Sciences, Isfahan, Iran

**Keywords:** antifungal, *Candida albicans*, colony count, nanocomposite, polymethyl methacrylate, strength

## Abstract

**Objectives** Polymethyl methacrylate as the most common material used in denture bases has some problems. The aim of this study was to introduce a new nanocomposite of PMMA to improve flexural strength and antifungal properties.

**Materials and Methods** In this experimental study, AgSiO _2_ nanoparticles were prepared, and their characteristics were confirmed by scanning electron microscope and energy dispersive spectroscopy techniques. Then the nanoparticles in the weight ratio of 0.1, 0.3, 0.5, and 0.7% were incorporated to heat-cured PMMA and the control group included no nanoparticles.

To measure the flexural strength before and after thermocycling three-point bending test was used. Eight samples per group with dimensions of 65 × 10 × 2.5 mm were used. Antifungal activity against *Candida albicans* (PTCC 5027) was investigated through colony count method. Statistical analysis was done by SPSS at significance level of *p* -value ≤0.05.

**Results** The mean flexural strength in groups 0.1, 0.3, and 0.7% was significantly higher than the control. After thermocycling flexural strength of the control group was significantly lower than 0.3 and 0.5% groups. As the concentration of nanoparticles increased the antifungal activity improved ( *p* < 0.05).

**Conclusion** Addition of nanoparticles AgSiO _2_ improved flexural strength and antifungal characteristics of PMMA.

## Introduction

Since 1940 1 polymethyl methacrylate (PMMA) has been the most common material used in denture bases due to its favorable characteristics including esthetic and ease of use and repair; however, there are some unfavorable characteristics such as low fracture resistance 2 3 and denture stomatitis. 4 Denture fracture was one of the most common reasons for denture repair. 2 3 Fractures happen more frequently in dentures since attachments limit the thickness of PMMA 5 6 and therefore make it more prone to fracture. There are some ways to reinforce PMMA such as the addition of fibers. 7 8 9 Further improvements have been made with nanoparticles. 10 11 12 13 14

The flexural strength has a crucial role in acrylic resins resistance to forces which are induced in oral environment, so evaluation of flexural strength is considered as one of the most important characteristics of mechanical properties of acrylic resin.

In some studies, adding different nanoparticles improved the strength of PMMA 11 12 13 14 and in some others had adverse effect due to accumulation of nanoparticles around each other. 11 14

Thermocycling which happens in oral environment every day can decrease mechanical properties of PMMA. Therefore, it was considered in this study. 15 16

Another problem associated with denture wearer is Candida denture stomatitis, which is caused by patient conditions and characteristics of PMMA which is a suitable media for fungal growth. 4

Different nanoparticles have proven to be antimicrobial agents such as AgSiO _2_ . 17 18 But the effect of these antimicrobial agents on mechanical aspects of PMMA is not clear.

The goal of this study was to introduce a novel nanocomposite of PMMA with both antifungal activity and improved mechanical properties. Accordingly, AgSiO _2_ nanoparticles were fabricated first and evaluated by physicochemical analysis. Then the nanocomposite of PMMA with different ratio of nanoparticles was fabricated and its antifungal (against *Candida albicans* ) and strength characteristics were assessed.

The null hypothesis was that addition of AgSiO _2_ to PMMA would not improve flexural strength and antifungal activity.

## Material and Methods

In this experimental study first, AgSiO _2_ nanoparticles were made, and added to four PMMA groups with proportions of 0.1, 0.3, 0.5, and 0.7%. In the control group no nanoparticles were added. Samples were evaluated for flexural strength and antifungal activity.

### Synthesis of AgSiO _2_ Nanoparticles

Sodium silicate liquid was diluted with distilled water with ratio of 1:4 and 1% of sodium dodecyl sulfate (SDS) was added. Temperature in this stage reached 90°C (PH of the solution was approximately 8 to 9 and stirring speed was 500 rpm). Temperature was lowered to 60°C and was stirred with speed of 200 rpm by a heat stirrer.

Sulfuric acid (concentration 98%) was diluted with distilled water in a ratio of 1:10 and based on stoichiometry the amount needed was provided. At this stage sulfuric acid solution was slowly added in 120 minutes to reach pH of 5.

Ten grams of 10% water solution of sodium chloride was added and stirred for 30 minutes. The heater was turned off and solution was gradually cooled while mixer was still working. 19 After a while, nano-silica particles were deposited which were washed several times with distilled water to remove impurities and were placed in 200°C furnace to dry.

0.2 g silica nanoparticles, 1% SDS, and 0.1 g of silver nitrate were added in 50 mL of distilled water and stirred at 80°C.

The equal equivalents of silver nitrate and hydrazine were added. After 30 minutes of stirring, the solution was placed into the oven and dried at a temperature of 200°C. After cooling, nano-silver silica powder by weight portion of one silver, two silica was prepared. 20 21

### Nanoparticles Characterization Test

The scanning electron microscope (SEM) (ZEISS, Germany) was used to study silver/silica nanoparticles and semiquantitative analysis of elements was done with energy dispersive spectroscopy (EDS).

### The Development of Nanocomposites

#### Specimens Preparation and Measurement of Flexural Strength

Four aluminum models with dimensions of 65× 10 × 2.5 mm were prepared and flasked. After setting of stone the flask was opened and aluminum dyes were removed and created space was replaced with heat-cured acrylic resin.

In the control group, heat-cured acrylic resin (Ivoclar, vivadent, Germany) was prepared according to manufacturing instructions and placed inside the empty space in the mold. After completion of polymerization, flask was opened slowly without stress. In this way acrylic resin samples were made.

In the case of nanocomposite samples, nanoparticle with the ratio of 0.1, 0.3, 0.5, and 0.7% weight for needed weight of acrylic resin was measured by digital scalar. In each group nanoparticles were dispersed by a magnetic stirrer into the acrylic resin monomer for 3 minutes, so even distribution of nanoparticles in resin matrix was achieved. The polymerization was done according to the manufacturer instructions. Samples were removed from the stone and thinned to appropriate dimensions by 450 grit sandpaper. Digital caliper was used to check the dimension of samples and those which were not appropriate were eliminated from the study. Samples were stored at 37°C for 50 hours.

The proper distribution of nanoparticles was investigated with SEM.

The flexural strength and deflection were measured for each group by using the loading machine (Walter testing machine, Germany) and with 3-point bending test and formula *S* = 3 *pl* /2 *bd* ^2^ in which *S* was flexural strength in Pascal, *p* was the amount of force exerted by the machine until breakage occurred in Newton, *l* was the distance between bases of the machine where force was applied, *b* was specimen width, and *d* was specimen thickness all in meters.

Sample from each group which had flexural strength closest to the mean of that group was chosen for microscopic examination.

#### Preparation of Specimens for Thermocycling

Thermocycled samples were prepared by abovementioned method. Thermocycling was done with temperatures ranging between 55 and 5°C and 5,000 repetition. In this way samples were placed in 5°C water for 30 seconds and then out of water for 10 seconds and 30 seconds in 55°C water. After completion of 5,000 cycles, flexural strength and deflection were measured as previously.

#### Preparation of Specimens for Evaluation of Antifungal Activity

Specimens with size of 10 × 10 × 2.5 mm were prepared. The methods and groups were as mentioned before. To assess antifungal activity, *Candida albicans* (PTCC 5027) was added to Sabouraud dextrose agar (Difco, Detroit, Michigan, United States) and incubated at 37°C for 48 hours. A suspension of 10 ^6^ *albicans* /mL was made through spectrophotometer (Biowave Biochrom, England) and wavelength of 530 nm.

Specimens were sterilized with UV waves for 5 minutes and then entered in a sterile tube containing 2 mL of fungal suspension. After incubation period of 90 minutes in 37°C the specimens were removed and were rinsed three times with distilled water for 1 minute. Then each specimen was entered in the tube containing 10-mL sterile saline and the adhered cells entered the saline by means of vortexing (Vortex mixer, United States). From this suspension, dilutions of 10 ^−1^ , 10 ^−2^ , and 10 ^−3^ in sterile saline were made. Then 0.1 mL of prepared suspension was entered into SDA medium and incubated at 37°C for 48 hours. The microorganisms were counted in CFU/mL. 22

Statistical analysis was done with SPSS software version 23 and by one-way ANOVA and Kruskal–Wallis test.

## Results

### Evaluation of AgSiO _2_ Nanoparticle

**Fig. 1:**
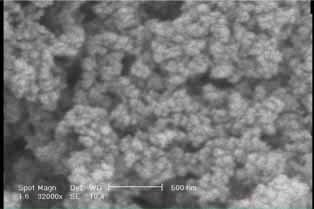
SEM illustration of AgSiO _2_ nanoparticles. SEM, scanning electron microscope.

The AgSiO _2_ nanoparticles were examined, and their size and quality were verified by SEM and EDS method. SEM images showed size of nanoparticles in the average range of 20 and 25 nm ( Fig. 1 ).

**Fig. 2:**
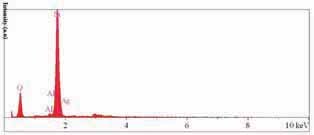
EDS illustration of AgSiO _2_ nanoparticles. EDS, energy dispersive spectroscopy.

EDS evaluation (in Kv 10.0, take off angle 35.0 degrees, and elapsed life time 30.0ms) of the three areas revealed the Hemogen distribution of silver and silica nanoparticles. Point check of nanoparticles showed the purity of silver/silica nanoparticles over 98% ( Fig. 2 ).

### Nanocomposites Microscopic Examination

**Fig. 3:**
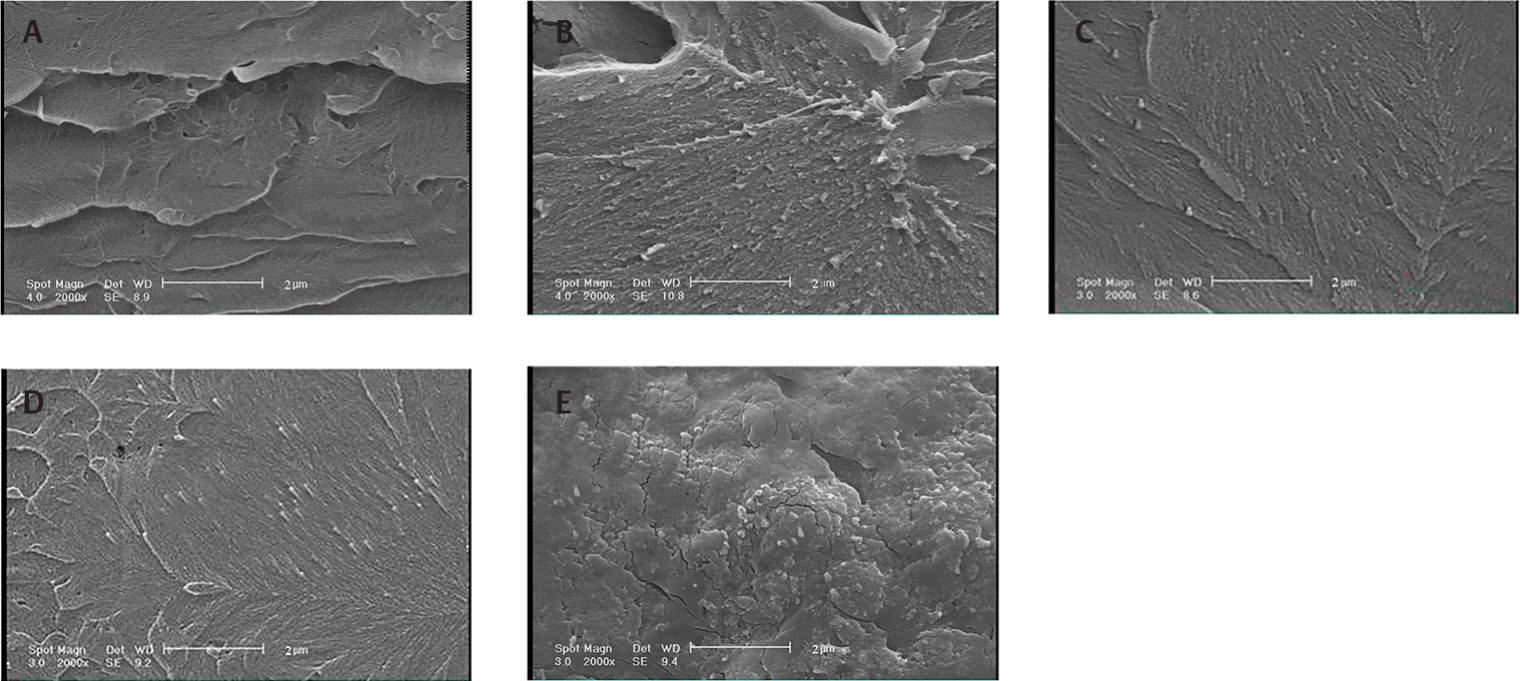
( **A** ) SEM illustration of acrylic resin. ( **B** ) SEM illustration of distribution of silver/silica nanoparticles in acrylic resin in group 0.1% nanoparticle. ( **C** ) SEM illustration of distribution of silver/silica nanoparticles in acrylic resin in group 0.3% nanoparticle. ( **D** ) SEM illustration of distribution of silver/silica nanoparticles in acrylic resin in group 0.5% nanoparticle. ( **E** ) SEM illustration of distribution of silver/silica nanoparticles in acrylic resin in group 0.7% nanoparticle. SEM, scanning electron microscope.

SEM images showed the distribution of silver/silica nanoparticles in acrylic resin which impacted on mechanical and other properties of acrylic resin ( Fig. 3 ).

The results of flexural strength and antifungal activity have been mentioned in the following.

### Results of Flexural Strength

**Fig. 4:**
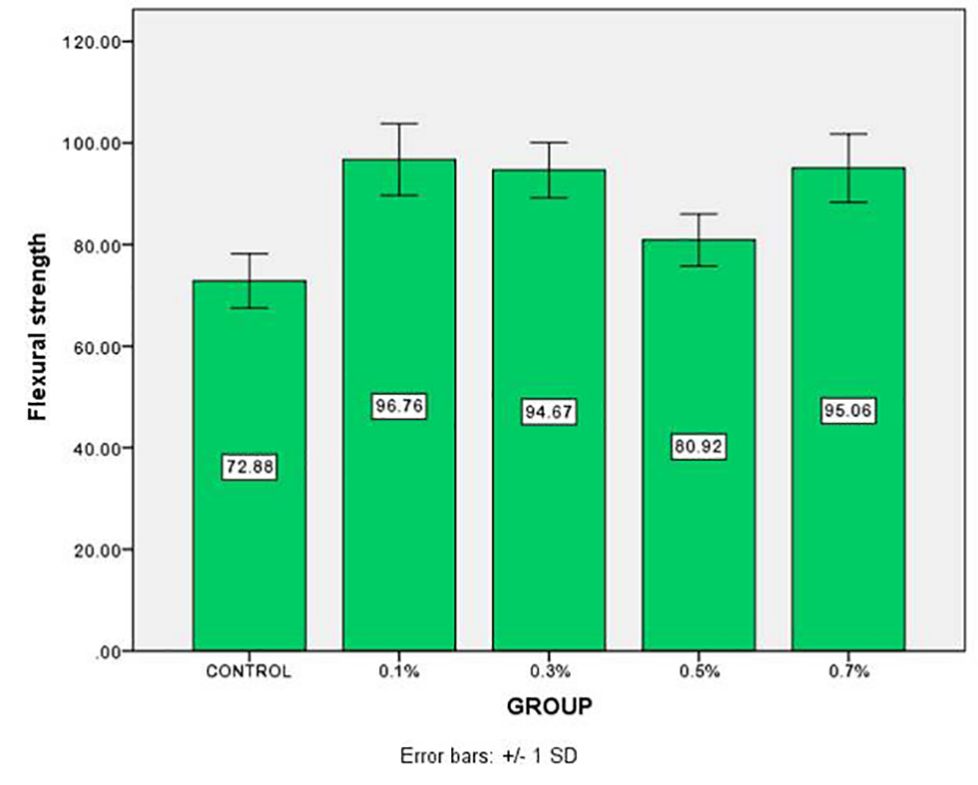
Profile of flexural strength.

One-way ANOVA and Tukey HSD analysis showed significant differences between the control group and 0.5% group with the other groups in such a way that the flexural strength of 0.1, 0.3, and 0.7% group was significantly higher than the control group and 0.5% group of the nanoparticles ( *p* -value <0.001). However, there was no significant difference between the control and 0.5% ( *p* -value = 0.07). The results of flexural strength are shown in Fig. 4 .

### Results of Flexural Strength after Thermocycling

**Fig. 5:**
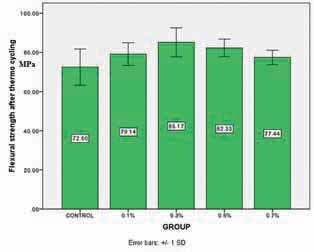
Profile of flexural strength after thermocycling.

Flexural strength of the control group after thermocycling was lower than the other groups. One-way ANOVA and Tukey HSD analysis showed this difference was statistically significant in 0.3% ( *p* -value = 0.003) and 0.5% ( *p* -value = 0.03) groups. The results of flexural strength after thermocycling are shown in Fig. 5 .

### Results of Antifungal Activity

**Fig. 6:**
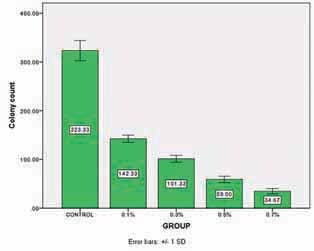
Profile of colony count.

One-way ANOVA and Mann-Whitney test revealed as the percent of nanoparticles increased the fungal colony count significantly decreased ( *p* -value = 0.05). The results of antifungal activity are shown in Fig. 6 .

## Discussion

As the elderly population is increasing there is an increase in need for dental prostheses. PMMA is one of the most widely used materials in fabrication of removable prosthesis. 1 The most common problem with it is its fracture during function. 2 Therefore, there is a need to improve its strength. Improvement in nanotechnology has led its way to the field of dentistry. In this study, it was tried to improve characteristics of PMMA by introducing a new nanocomposite. The hypothesis that addition of AgSiO _2_ to PMMA would not improve flexural strength and antifungal activity was rejected based on the results.

Although patient hygiene has an important role in the inhibition of denture stomatitis, but in very old or immune-deficient patients there is a need to inhibit microorganism growth. It has been proven that nanotechnology can provide antimicrobial effects. 17 18 It was important to use a nanoparticle with antimicrobial effect without disadvantage for mechanical properties. Since the flexural strength has an important role in acrylic resin resistance against forces exerted on prosthesis in the mouth, evaluation of flexural strength is one of the most important factors in acrylic resins. Therefore, in this study flexural strength was chosen to measure the mechanical properties.

To use or not to use other auxiliary factors to bond nanoparticles with acrylic resins was another issue that had to be decided upon. In some studies, adding coupling agents to improve more effective bond between the filler and acrylic resin was emphasized. For example, in a study adding only 0.1% of the coupling agent showed favorable results on mechanical properties, but more concentrations resulted in poor mechanical properties. However, the effect of humidity on the bond strength in long term was not considered. 23

A review article on silane coupling agents reported that it will eventually disintegrate in the mouth environment. 24

In another study the relationship between the amount of silane on filler and the weakening effect of mechanical properties after immersion in water was demonstrated. 25

In this study, nanoparticles were conditioned with methacrylic acid which is one of the ingredients of heat-cured PMMA monomer, as a coupling agent. This method was used by Khaled et al 26 for synthesis of TiO _2_ −PMMA nanocomposite. So, the nanoparticles were ready to bond with polymer matrix. With this method successful bond between nanoparticles and polymer matrix was made without disadvantages of external silanes.

In some studies, addition of nanoparticles improved flexural strength of the acrylic resin 11 12 13 14 and in some others, it had negative effects due to the accumulation of nanoparticles around each other. 11 14 Even distribution of the nanoparticles in acrylic resin, depends on stirring rate, period of stirring, and temperature. The more uniform distribution of nanoparticle causes more uniform physical, mechanical, and biological properties. In this study magnetic stirrer as an available specified device was used for a certain time to decrease nanoparticles aggregation and provide conditioning and dispersion of nanoparticles in the monomer.

From each group, the sample which was closest to the average flexural strength of that group was selected to be studied microscopically by electron microscopy to have better interpretation of the observed properties. In this study, addition of nanoparticles improved flexural strength, which was statistically significant in groups 0.1, 0.3, and 0.7%. The SEM investigation showed that in 0.1 and 0.3% groups light distribution of nanoparticles was obtained but in 0.5 group accumulation of nanoparticles was observed. In 0.7% group heavy but even distribution of nanoparticles was observed. This distribution explained why in 0.5% group the strength was reduced.

Thermocycling can be expected in the stressful environment of the mouth and happened in everyday life. In previous studies the negative effect of this phenomenon on acrylic resin has been reported. 15 16 In the present study, flexural strength improved by adding nanoparticles even after thermocycling in all groups, but this improvement was statistically significant in 0.3 and 0.5% group compared with the control group. It seems that thermocycling can degrade polymer’s bond, so it is probable that in 0.7% group more bonds were degraded than others with lower bond counts. In 0.1% group less amount of bond was formed from the beginning and from this amount, some were degraded due to thermocycling stress.

Another area to consider is the effect of addition of these nanoparticles on color of samples. Since AgSiO _2_ nanoparticles had relatively dark shade in acrylic resins, especially in the 0.7% group, if the main purpose is to improve mechanical properties, it may be better to use nanoparticle in lower concentrations and if there is a need for most antifungal activity, dark shade should be covered with some proper pigments.

The limitation of this study was performing it in the experimental situation. Also, it is suggested to evaluate biocompatibility and some other mechanical properties in another study.

## Conclusion

Addition of nanoparticles AgSiO _2_ to PMMA led to an increase in flexural strength; the best results were seen in 0.1, 0.3, and 0.7% nanoparticles groups.

Addition of nanoparticles AgSiO _2_ to PMMA led to increase in flexural strength after thermocycling and the best results were seen when 0.3 and 0.5% nanoparticles were added.

Addition of AgSiO _2_ improved antifungal activity significantly.
